# Sedentary behaviour in rheumatoid arthritis—barriers and facilitators, a qualitative analysis

**DOI:** 10.1093/rap/rkag137

**Published:** 2026-10-30

**Authors:** Florentia Kitas, Carolyn Greig, Matthew Willett, Jet Veldhuijzen van Zanten, Sally A M Fenton

**Affiliations:** School of Sport, Exercise and Rehabilitation Sciences, University of Birmingham, Edgbaston, Birmingham, UK; NIHR Birmingham Biomedical Research Centre, University Hospitals Birmingham and the University of Birmingham, Birmingham, UK; School of Sport, Exercise and Rehabilitation Sciences, University of Birmingham, Edgbaston, Birmingham, UK; NIHR Birmingham Biomedical Research Centre, University Hospitals Birmingham and the University of Birmingham, Birmingham, UK; School of Sport, Exercise and Rehabilitation Sciences, University of Birmingham, Edgbaston, Birmingham, UK; Centre of Precision Rehabilitation for Spinal Pain, University of Birmingham, Birmingham, UK; School of Sport, Exercise and Rehabilitation Sciences, University of Birmingham, Edgbaston, Birmingham, UK; NIHR Birmingham Biomedical Research Centre, University Hospitals Birmingham and the University of Birmingham, Birmingham, UK; Centre for Movement and Wellbeing (MoveWell), University of Birmingham, Birmingham, UK; School of Sport, Exercise and Rehabilitation Sciences, University of Birmingham, Edgbaston, Birmingham, UK; NIHR Birmingham Biomedical Research Centre, University Hospitals Birmingham and the University of Birmingham, Birmingham, UK

**Keywords:** rheumatoid arthritis, sedentary behaviour, barriers, facilitators, qualitative analysis

## Abstract

**Objectives:**

Sedentary behaviour (SB) is a modifiable risk factor in the management of rheumatoid arthritis (RA). SB interventions may be more acceptable where pain, fatigue and fluctuating disease activity limit physical activity (PA). To inform the development of effective/scalable interventions, this study aimed to: (1) determine how people with RA conceptualize SB and (2) explore lived experiences of SB to identify barriers and facilitators.

**Methods:**

Semi-structured interviews were conducted with adults with RA, recruited in the UK using opportunistic and snowball sampling. Interviews were audio-recorded, transcribed verbatim and analysed using inductive thematic analysis.

**Results:**

Participants (*n* = 14) used SB, activity, movement and PA/exercise interchangeably. Three themes were generated. Motivation explained engagement or breaks in SB; SB was reduced when movement was meaningful, enjoyable or linked to valued roles. Anticipated effort and fear of pain reinforced SB. Resilience and adaptive strategies reduced SB, while competing demands reinforced SB. Movement, captured negotiation between activity and rest, shaped by physical symptoms, contextual factors and sociocultural influences. The same factors (e.g. family) operated as a facilitator and barrier. Participants recognized that rest prevented exacerbations, however, prolonged sedentariness worsened stiffness and function. Identity in Flux, reflected changes in perceived capability, stigma and loss of identity reinforcing SB for some, while others reconstructed identity to maintain valued roles and social participation.

**Conclusion:**

SB is shaped by psychological, social and contextual determinants, not symptoms alone. Interventions should adopt identity-sensitive, flexible approaches that enhance meaningful motivations and address pain-related fears, rather than relying on prescriptive targets.

Key Messages

- Sedentary behaviour in rheumatoid arthritis is meaningful, adaptive daily behaviour extending beyond symptom variability alone.
- RA sedentary behaviour interventions should address motivation, identity, symptoms, context, autonomy and meaningful sedentary activities.

## Introduction

Rheumatoid arthritis (RA) is a chronic, systemic autoimmune condition that primarily targets the joints, causing pain, swelling and stiffness [1]. RA affects major organs and systems, including the heart, blood vessels, lungs and metabolic system, and increases infection risk [1]. People with RA have an elevated risk of developing mental health issues, e.g. depression and anxiety, contributing to diminished quality of life (QoL) [2–4].

RA can be managed through lifestyle modifications (e.g. changing diet, reducing stress and increasing physical activity (PA)). The European Alliance of Associations for Rheumatology (EULAR) recommends PA as a core component of RA management [5–8] and have highlighted the potential role of sedentary behaviour (SB).

SB is defined as ‘*any waking behaviour characterised by an energy expenditure of ≤1.5 Metabolic Equivalents (METs) and a sitting or reclining postur*e’ which includes activities such as watching television, using a computer, reading or driving [9]. Epidemiological research suggests SB in the general population is associated with increased all cause, cardiovascular and cancer mortality [10–12]. Experimental research suggests inflammation as a mechanism underlying these processes in SB [13]. It has been hypothesized that SB may exacerbate the already heightened local and systemic inflammation in RA and therefore may have adverse consequences for RA outcomes [14]. In response, research in RA started to explore the role of SB for clinical and patient important outcomes.

Research (mostly cross-sectional) exploring SB in RA indicates higher levels of SB are associated with higher disease activity, increased pain, lower physical function and elevated risk of CVD [13, 15–18]. As such, lifestyle interventions to reduce SB in RA could improve RA outcomes. SB interventions may also encourage overall movement in people living with RA, given that most of the traditional advice around movement and PA for RA has been focused on moderate-to-vigorous (MVPA) PA (i.e. ≥ 3 METs) [5]. Engagement in MVPA can be challenging for people living with RA, especially during periods of disease flare-ups, or for those with limited physical function and chronic pain or fatigue [19, 20]. Reducing SB may offer an alternative avenue for PA promotion, potentially deemed more acceptable and feasible in the context of these symptom-related challenges.

For lifestyle interventions to support behaviour change, they should reflect the needs and preferences of users. Thus, an SB intervention in RA should be grounded in an in-depth understanding of the experience, perspectives and psychosocial context of people with RA to address key behavioural challenges to reducing SB, i.e. perceived barriers and facilitators. Qualitative research captures feelings, beliefs and values that motivate behaviours [21]. To date, only one qualitative study has explored SB determinants in RA [20]. Participants were largely functionally independent though some had disabilities in activities of daily living. SB was influenced by symptoms fluctuations, (i.e. pain and/or fatigue), served as a strategy for managing daily life (e.g. sitting as a break between activities) and reflected lifestyle patterns. Therefore, further research exploring how individuals with RA perceive and experience SB—considering personal beliefs, values, cultural norms, self-regulation, motivations and autonomy—will deepen our understanding of its psychosocial context and determinants.

The aim of this study was therefore to explore the experience of SB in people with RA, integrating questioning around deeper psychosocial, cultural and identity-related dimensions, to identify key determinants of SB that can be targeted by interventions. Specific objectives were to: (1) determine how people with RA conceptualize SB and (2) explore their in-depth experiences of SB, considering their understanding, attitudes, beliefs and mental health, to identify the barriers to, and facilitators to reducing or interrupting SB.

## Methods

### Theoretical framework

This study was grounded in phenomenology, with the aim of understanding participants’ lived experiences of SB in RA. Interview data were analysed using template analysis. Findings are reported using the COnsolidated criteria for REporting Qualitative research (COREQ) guidelines [22] (Supplementary Data S1, available at *Rheumatology Advances in Practice* online).

### Participant selection

Adult participants (≥18 years) with a self-reported diagnosis of RA and ability to communicate in English were invited to take part and were contacted through email. Eligibility was confirmed against predefined criteria prior to obtaining informed consent. Following consent, participants provided the contact details of their treating consultants to confirm their diagnosis where possible. All participants gave informed consent, and ethical approval was granted by the University of Birmingham Research Ethics Committee (ERN_1856-May2024).

### Sampling

Opportunistic sampling was used to recruit people with RA who participated in a previous ecological-momentary assessment study, which aims to classify types of PA and SB using machine learning models among people with RA, at the University of Birmingham (Research Ethics Committee reference: 22/LO/0638) (*n* = 7). To increase diversity, participants were also contacted by email through RA community/support groups across the UK, using snowball sampling (*n* = 7). Group representatives participated first and subsequently shared the study with other group members. A sample of 10–15 participants was anticipated, based on recommendations for achieving data saturation [23].

### Data collection

Semi-structured, one-to-one, 1-h interviews were undertaken by the lead researcher (FK, MSc, Post-graduate Doctoral Researcher, female). FK had no previous experience of conducting qualitative interviews but had received theoretical training in qualitative research and interview methods during her undergraduate and postgraduate degree. Interviews took place from April 2024 to February 2025. Eleven interviews were conducted via Zoom, one took place in person, and two were conducted over the phone. Zoom interviews were video- and audio-recorded using the Zoom platform, and in-person and phone interviews were audio-recorded using a recording device. Transcripts were offered to participants at the end of the interviews. Socio-demographic details were collected before the interview. Participants had not met FK before the interview.

The interview guide (Supplementary Data S2, available at *Rheumatology Advances in Practice* online) was developed by the research team (FK, SF and CG) with input from a Patient and Public Involvement (PPI) group, who helped shape the questions to use more lay terminology (i.e. change ‘SB’ to ‘sitting behaviour’) and be less leading (i.e. change ‘Do you do X out of necessity?’ to ‘Why do you do X?’), suggested to provide a definition of SB, and include one question around mental health. A flexible approach to interviews was maintained and any new SB-related topics that evolved during interviews were discussed. Authenticity of the data was maximized by the researcher providing a neutral introduction of the study aim, information that this research was conducted as part of her PhD studies, emphasized confidentiality and anonymity and avoidance of leading questions. Through comparative analysis across chronological interview batches, FK tracked the emergence of new codes. By the eleventh interview, no new codes were generated, and a final 3 interviews served as a confirmation buffer largely reinforcing and refining the existing template. Saturation was deemed complete when further data collection matched/were similar to existing testimonies surrounding the core themes. Participants did not provide feedback on their transcripts.

### Analysis

Interviews were transcribed verbatim by FK and were re-checked for errors by re-listening to the audio-recording. NVivo software (version 15) was used to facilitate systematic structuring of data and audit trial of decisions and data management. Interview data were analysed by FK, with input from MW and SF. FK read each interview transcript and listened to the interview recordings to support familiarization.

#### Objective 1

Participant responses to the first interview question (i.e. ‘What is your understanding of sedentary behaviour?’) were extracted and tabulated to allow for systematic comparison, identifying similarities and differences in interpretation across the sample.

#### Objective 2 (template thematic analysis)

Interview data were analysed using a template analysis approach, applying thematic analysis. First interview data from the first four transcripts were inductively coded by the lead researcher (FK) following guidance by Braun and Clarke [24]. The codes (with relevant quotations) were organized into preliminary themes/sub-themes, to create a coding template. Individual extracts of data were coded in as many different themes as they fit and as many times as deemed relevant.

To develop the coding template, supporting quotations were written on individual post-it notes to generate a mind-map, enabling visual exploration of patterns and flexibility of the thematic analysis. FK, SF and MW collaborated to ensure alignment between the data and emerging themes over a series of in-person/video conferences to examine evolving thoughts and ideas during the coding process. Accounts or codes that departed from the dominant coding were not ignored and were retained in separate free nodes on NVivo to ensure they weren’t lost. During these peer debriefs, coded data extracts were reviewed to determine whether a coherent pattern was apparent. Themes or subthemes that were too similar were collapsed, and the research team ensured that themes were discrete and broad enough to capture a set of ideas in participant accounts.

After four interviews, the thematic framework and coding template were reviewed and discussed within the research team before being applied to subsequent transcripts [25, 26]. Consistent with template analysis, the coding template was not treated as fixed; rather, it was iteratively challenged by the research team and refined throughout analysis. The remaining interviews were also coded inductively to evolve the framework and generate as many in-depth insights as possible.

Any new emerging codes or themes from following transcripts were added to the template where appropriate, existing codes were modified or collapsed, and relationships between codes were reviewed as understanding of the dataset developed. FK kept a reflexive diary to document evolving thoughts/assumptions, coding decisions, interpretations and evolving understandings of SB in RA. These reflections were discussed regularly with co-authors to challenge assumptions and enhance analytical rigour. This helped to ensure that physical barriers such as pain and fatigue were not underestimated or overemphasized, shifting from viewing SB as a lifestyle choice, but as a potential, fluctuating strategy for pain/fatigue management. Notes were made after each coding session and team discussion, capturing analytic decisions and emerging questions. Through this iterative process, the final thematic structure was generated from the complete dataset. Final themes were then organized and are presented according to barriers and facilitators to reducing or interrupting SB. Findings of the study will be shared with participants following publication.

## Results

### Participants

All 14 participants (*n* = 9 females) who were approached agreed to participate. Interviews lasted 60–90 min (media*n* = 75 min). Participants identified as White British (*n* = 11), Black British (*n* = 2) or Asian (*n* = 1). The mean age was 63.07 years (SD = 12), with symptom durations between 2 and 62 years. Further demographic details can be found in Table 1.

**Table 1:** Participant demographics.

Participant no.	Gender	Age	Ethnicity	Year of presenting symptoms	Employment status	Education	Living arrangements
1	M	41	Asian	2023	Full-time	Doctorate	With partner and child
2	F	74	White British	1974	Retired	Bachelor’s degree or equivalent	With partner
3	F	63	White British	2017	Retired	Master’s degree or equivalent	With partner and child
4	F	81	White British	1963	Retired	Bachelor’s degree or equivalent	With partner
5	F	72	White British	2001	Retired	Other—post-graduate diploma	With partner
6	M	63	White British	2013	Part-time	Other—City and Guilds	Single
7	F	61	White British	1985	Retired	Secondary school	With partner
8	F	61	Black British	2003	Retired	Other—Technical College	With partner
9	M	79	White British	1981	Retired	Master’s degree or equivalent	With partner
10	F	42	White British	2011	Full-time	Bachelor’s degree or equivalent	With partner and child
11	F	64	White British	2011	Part-time	Bachelor’s degree or equivalent	With partner
12	M	58	White British	2022	Full-time	Master’s degree or equivalent	With partner and child
13	M	70	White British	2004	Retired	Bachelor’s degree or equivalent	With partner
14	F	54	Black British	1984	Part-time	Bachelor’s degree or equivalent	Single

### Objective 1

Participants provided various descriptions of SB (Table 2). While several participants identified SB as time spent sitting or lying down with low energy expenditure as per the accepted definition [9], others equated it more broadly with being physically inactive or the opposite of being physically active.

**Table 2:** Participants’ conceptualization of sedentary behaviour.

Participant No.	Participant interview responses
RA01	*‘Sedentary, it’s something like someone like not being active and then somebody who don’t go out and then do some activity, I think.’*
RA02	*‘Well, it’s just behaviour where you will be sitting down a lot and not moving very much and not doing many activities really.’*
RA03	*‘There’s loads of research and findings that people who are more physically active just have better health and more longevity. Um, and it’s better for your bone strength, muscle strength, your strength in general. Um, enjoyment of life, vitamin D, um digestion, sleep, everything. There -, were made to be active and the problem is that lots of people are doing things that are all about sitting down.’*
RA04	*‘Sedentary behaviour is actually um really, really important for the simple reason that if you are sitting around a lot or you know, and not moving an awful lot, then you’re going to, with rheumatoid arthritis you actually end up seize.’*
RA05	*‘Eh, well-being, eh fairly bonile, I suppose, and not getting up off your rear end more frequently than you have to.’*
RA06	*‘Um, sedentary it’s kind of like, how I understand it, if I was to go somewhere, say for example, out for a movie in a cinema and then I sit there and watch a movie and then I can’t get up later. To me that’s sedentary behaviour.’*
RA07	*‘Uh, sedentary behaviour is just sitting about, not doing very much.’*
RA08	*‘I know sedentary, sedentary behaviour is not great for anybody in regard to whether, in respect to whether they got rheumatoid arthritis or not. It’s you know, for your health and um your wellbeing, you should, um, move. Uh, more people should move than they do. You know so sedentary behaviour can lead to um, you know, people gaining weight and all of this sort of stuff… So that’s, in a nutshell, kind of what I know that what happens if you just sit still too long.’*
RA09	*‘Well, I guess it means, sedentary means sitting down being um nonactive and just basically not, not doing any sort of activity or whatever.’*
RA10	*‘So, I’m guessing it’s kind of um, just sitting around on the sofa. I guess actually when you’re working at a desk, you’re sedentary as well so it’s basically not much movement.’*
RA11	*‘It’s behaviour where you’re doing a lot of sitting. Not using your muscles very much at all. It’s kind of being a bit the couch potato.’*
RA12	*‘So, sedentary behaviour means uh, comes from the Latin related to sitting down and presumably broader context you refer to this kind of inactivity.’*
RA13	*‘I presume sedentary is all to do with when you’re sitting, you’re, how you behave when you’re either in a chair or you’re lying down, as opposed to the opposite which is active behaviour.’*
RA14	*‘It’s to do with basically how much you sit down, how inactive you actually are, as well…’*

Throughout the interviews, participants commonly expressed a desire to be ‘less sedentary’ by engaging in more PA or exercise, and the terms *SB*, *activity*, *movement* and *exercise* were used interchangeably. This influenced how participants discussed their everyday practices, values and identities in relation to movement, blurring the boundaries between reducing SB, being more physically active, and exercising for health/enjoyment. This posed challenges for interpretation, the thematic analysis that follows reflects these complexities. Participants’ motivations to reduce SB may have been confused with motivations to increase activity, therefore this analysis captures broader values, intentions and perceived benefits that may shape participant desires to sit less. Further, perceptions of movement were often not in reference to specific behavioural constructs of PA or SB; thus, participants lived experience of movement is captured along a continuum rather than treating SB and PA as behavioural opposites. Where a participant directly referred to either behaviour, this was explicitly mentioned; otherwise, this ambiguity was more broadly referred to as ‘movement’ in the below analysis. Lastly, participant accounts of SB were also embedded within broader perceptions of themselves as ‘active’ or ‘inactive’ people, often informed by previous exercise, occupational and lifestyle experiences; therefore, interpretation in this analysis considers SB within participant’s broader narratives of changing health, function and sense of self. Each theme captures a different dimension of how participants make sense of ‘movement’—comprising SB, activity and exercise—in the context of living with RA, while acknowledging that these behaviours are conceptually intertwined and experienced as part of a broader narrative of managing health and self.

### Objective 2

Three higher-order themes were generated: (1) *Motivation*, (2) *Movement* and (3) *Identity in Flux—*each with associated subthemes. The following sections describe these themes, and supporting quotes that capture participants lived experiences are outlined in Table 3. Figure 1 displays a conceptual model illustrating the identified themes.

**Figure 1:**
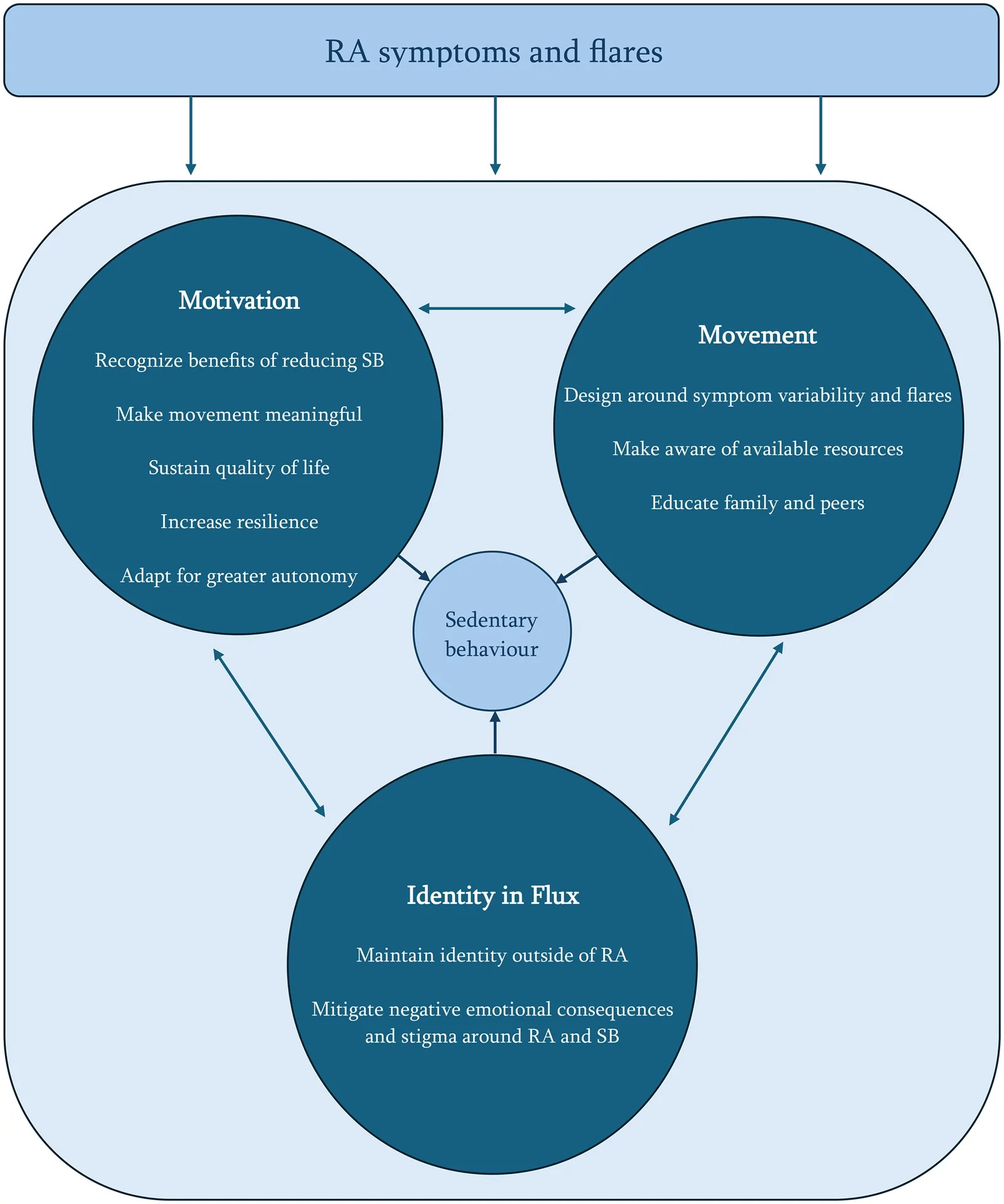
Conceptual model of themes

**Table 3:** Themes, subthemes and supporting quotes.

**Theme 1: Motivation**		
**Subtheme**	**Facilitator**	**Barrier**

***Recognizing benefit, anticipating struggle***	***RA08:*** *‘I’m motivated to keep my condition under control… it’s allowed me to do virtually 99% of what I need to do in life without any hinderings.’*	***RA14:*** *‘I think part of it is set in my mindset now because the energy and effort it takes. And then how slow I am… I just don’t want to go through that, that struggle because my body is already aching. In my mind I think, ‘Oh it’s just going to hurt even more’, which is silly really because exercise will help, but it’s like a lot of mind block on it.’*
***The paradox of meaningful activity***	***RA08:*** *‘It’s a mindset… I think I’m of the right mindset. I know not everybody likes exercise and for some people it’s a bind, it’s a chore. It’s never a chore for me, it’s something that I like to do, and that’s me.’*	***RA05:*** *‘Well, the fact that most of my hobbies don’t involve exercise…the book group is sitting around the table discussing books, playing bridge is something you do sitting down…even if you meet friends for lunch or coffee… it tends to be sitting eating or drinking…So, our hobbies don’t tend to encourage activity.’*
***Sustaining quality of life and competing obligations***	***RA02:*** ‘*Well, I think because I can see that if I don’t keep going then I could restrict my life quite a lot and I like to be able to go out and see people. I like to be able to go to places, different places, travel… go out and meet my friends… And I can see that if I don’t keep pushing through, it’s going to get harder and harder for me to do things…and then I’ll be stuck by myself inside.’*	***RA12:*** *‘I think, yeah, to take you back to Ground Zero…I cease to derive any enjoyment out of work at all, but I still have to do it… I think I need to re-map my life. I need to redefine, re-build, re-evaluate my routine, my relationship with my wife, and what I’m going to fill my days with… I think I need to reinvent myself really.’*
***The spectrum of resilience***	***RA09:*** *‘I’ve always been lucky in that I’ve always, you know, (been) a positive person… most of the time I just feel grateful and lucky that I can do what I can do.’*	***RA14:*** *‘I accepted that, that’s what my life is now…I rely on people … I think it took me a very, very long time…to actually accept my limitations and I think after accepting them… I don’t think I’ll fight as hard against as I did before… there’s a lower expectation from me because of the arthritis… sometimes that gets you kind of like, ‘Well I don’t need to do it then.’… I mean a lot of what I do is… sitting down, not physically getting up and doing.’*
***Adaptive strategies to maintain autonomy***	***RA02:*** *‘I’m not disabled in my own house, but I am in other peoples, because everything is set up different. I have my house set up so I can manage things …. But when I go somewhere else… I can’t make up myself food…Or even make myself a drink …So that does make actually quite a lot of difference.’* ***RA04:*** *‘After all these years of having rheumatoid arthritis, you get to actually organising your life very much better than you would normally… So … things that I’ve already cooked are in the freezer. So therefore, it doesn’t matter if you’re feeling very bad or really struggling, there’s always food there.’*	***RA03:*** *‘I also know it’s really easy to let time drift and suddenly know what the time really is…I know I can sit still for three h…And I can be engrossed.’*

**Theme 2: Movement**		
**Subtheme**	**Facilitator**	**Barrier**

***Symptom-driven movement and flare cycle***	***RA11:*** *‘If I’m having a flare, I have to remind myself that my joints will thank me in the long run if I tried to move them, and by being sedentary… I will probably seize up more.’*	***RA02:*** *‘Unpredictability, because you don’t know when a flare up is going to come… So, if you were to sign up for something like a course that you were to pay each term for, you might be reluctant because you might feel like you might not be able to do half of it.’*
***Contextual influences on movement and sedentary behaviour***	***RA02:*** *‘I’ve got this strange physio app… and I have to try and do that every day… I volunteer… and I’ve got to be able to walk little distances so that I can meet people… I want to do things like help my daughters with the grandchildren… all those things are actually helping me to keep active.’*	***RA10:*** *‘I guess it’s really not feeling in control… the rheumatologist has the kind of control over that (medication), so you’re kind of at their mercy of what, what they’re going to offer you.’*
***Social influences on movement and sedentary behaviour***	***RA02:*** *‘It’s quite important really for the person that lives with you to be understanding but not to be over caring… he knows that I always want to be independent…so it’s actually good to the point of me having to say to him, “Look can you help me with this”, because he doesn’t offer help. But that’s good in a way because it makes me do things.’*	***RA14:*** *‘I did find, and I still do, to a degree, that my family can also be my worst enemy too, in that I want to do things, and I can do it, but they want to take over and do it because I’ve got the arthritis…there’s a fight, and always has been from my independence.’*
**Theme 3: Identity in Flux**		
**Subtheme**	**Facilitator**	**Barrier**
***Changing self-image***	***RA11:*** *‘I’ve got friends now that I wouldn’t have had. I’ve got a really good relationship with my rheumatology department and one of my consultants asked me to get involved in some projects with the BSR and all those sorts of things I could never imagine doing before I had RA.’*	***RA02:*** *‘Well since being diagnosed… it’s just more that I just couldn’t do things so, so I had to change, it was kind of forced upon me.’*
***Emotional consequences***	***RA07:*** *‘I don’t know, I’m sure it’s just psychological, but I do feel like I’ve achieved something… I do feel better for it, which is obviously why I do it, I think.’*	***RA13:*** *‘If at work…they say, “Let’s all go to the pub for a drink after work”, I’m actually worrying about it, because I know I can’t stand at the bar, I have to sit down and because, rightly or wrongly, I’ve made decision not to discuss my illness with people at work, I had to manipulate situations. And my way of manipulating it was to make sure I walked into the pub first… and say, “Oh let’s go over there, there’s some space”, and consciously choose a place where I could sit down.’*
***The social cost of RA***		***RA04:*** *‘When you’ve got long-term health problems and you get so isolated… people get fed up with you not being able to jump and go and… you have to plan long-term or rest and all this kind of thing before you can do anything. You know, it’s quite difficult.’*

#### Theme 1: Motivation

Motivation was central to understanding SB experiences in people with RA, shaped by health intentions, personal values, and challenges including symptoms, and emotional strain. Participants described both facilitators and barriers to motivation to be less sedentary, with some demonstrating resilience through adaptive strategies that supported motivation and maintained independence.

##### Subtheme 1.1: Recognizing benefit, anticipating struggle

*Facilitator:* Participants recognized the benefits of movement, including better health, higher energy levels, better management of symptoms (pain, fatigue and seizing of joints), weight control and maintaining strength. Viewing movement as self-care motivated many to overcome barriers and sustain movement, reducing SB.

*Barrier:* Despite recognizing benefits, symptoms of pain, fatigue and low mood diminished motivation to engage in movement. Anticipated effort, pain and slowness, created a sense of futility, undermining motivation to move more, reinforcing SB. Participants avoided small opportunities to break sedentary patterns, such as making themselves a drink.

##### Subtheme 1.2: The paradox of meaningful activity

*Facilitator:* Participants aligned movement with personal values, associated to their culture and upbringing. Movement was a byproduct for motivation to engage in meaningful activities (e.g. going to the gym or on walks were described as hobbies). Pleasure and interest were dominant to retaining engagement in more active activities, reducing SB.

*Barrier:* Many valued hobbies were naturally sedentary (reading, playing bridge). Motivation was not solely a matter of willpower, but also contingent on the nature of activities individuals prioritized.

##### Subtheme 1.3: Sustaining quality of life and competing obligations

*Facilitator:* Participants articulated both movement and/or being less sedentary as a means to fulfilling their dreams of travel, socialization and avoiding isolation. Movement supported relationships with family and social connection. Efforts to reduce SB lowered the risk of declines in functional capacity and living a restricted lifestyle. Movement and minimized SB were framed as a mean to conserving quality of life and social participation.

*Barrier:* For some participants, competing demands such as employment constrained their ability to prioritize movement and/or reduce their SB e.g. when working at a desk, making broader life goals difficult to pursue.

##### Subtheme 1.4: The spectrum of resilience

*Facilitator:* Resilience sustained motivation to reduce SB and/or increase movement, preventing physical symptoms from overwhelming their mental health. Adopting a ‘just get on with it’ mindset to resist engaging in SB in times of pain or fatigue, and focusing on what remained possible, rather than what was lost due to RA, challenged perceived limitations in levels of movement. Acceptance of limitations, proactive engagement with life, and efforts to increase movement or reduce SB, enabled agency and self-efficacy in the management of their disease.

*Barrier:* For some, acceptance of their diagnosis and limitations was accompanied by a reduction in the intensity with which they fought against their symptoms. Emotional exhaustion, lowered social expectations and physical limitations legitimized sedentariness and diminished the drive to resist it.

##### Subtheme 1.5: Adaptive strategies to maintain autonomy

*Facilitator:* Assistive devices and home modifications reduced barriers, enabling daily functioning, movement and agency, reducing experiences of disability. Proactive planning, e.g. preparing food in bulk, managed fluctuating symptoms. Being organized was described as a necessity to reduce anxiety and support independence, creating opportunities to break SB.

*Barrier:* Entrenched routines and longstanding sedentary habits reduced motivation to change. Retirement and unstructured days often encouraged prolonged sitting, making movement less intentional. SB was a product of everyday rhythms and internal narratives that shaped how participants organized their days.

#### Theme 2: Movement

Movement, captured how participants balanced activity and rest, highlighting the interactions between an unpredictable body and the surrounding environment. Physical symptoms, contextual factors and sociocultural influences shaped daily routines, enabling or constraining movement.

##### Subtheme 2.1: Symptom-driven movement and the flare cycle

*Facilitator:* Pacing and listening to the body were central to engaging in movement. Adjusting activity and increasing SB prevented flares or joint damage, balancing exertion with rest. However, while flares forced reductions in activity, participants recognized a cycle in which enforced sedentariness led to stiffness and functional decline, reinforcing the need for feasible movement. This balance was learnt through experience.

*Barrier:* Movement was dependant on pain, fatigue, discomfort, stiffness, seizing or swelling. Comorbidities further increased SB and dependence. Long-term joint damage and foot deformities were also major barriers. During severe flares, activity was reduced to minimal movement within the home and avoidance of basic tasks (e.g. opening a jar, turning on a light switch). Flare unpredictability limited participation in structured or paid activities.

##### Subtheme 2.2: Contextual influences on movement and sedentary behaviour

*Facilitator:* Interpersonal, environmental and healthcare factors influenced SB reduction. Responsibilities (e.g. dog ownership, caring for children/grandchildren) created routine movement. Access to gardens, parks or rural environments were ‘natural enablers’, encouraging incidental activity without formal exercise. Effective medication, surgery or improved symptom control further enabled movement.

*Barrier:* Bad weather, transport difficulties or lack of RA-tailored classes reduced opportunities to be less sedentary. Caring for a family member with illness/disability reduced independent mobility, increasing SB. Healthcare factors constrained movement; often reporting limited advice on activity, and ineffective or changing treatments reduced perceived control. This created a sense that physical capacity was contingent on medical decisions.

##### Subtheme 2.3: Social influences on movement and sedentary behaviour

*Facilitator:* Encouragement from family and friends enhanced motivation, confidence and independence. Exercising or walking with others was preferred to being active alone. Participants emphasized the importance of support that maintained autonomy.

*Barrier:* Over-caring from relatives or friends limited opportunities to engage in non-sedentary behaviours and reinforced dependency. While support was valued, excessive interference or involvement could unintentionally discourage movement.

#### Theme 3: Identity in flux

Identity in Flux reflected how RA reshaped participants’ sense of self and influenced movement. Shifts in self-image, feelings of reduced capacity and emotional consequences contributed to avoidance, reinforcing SB, while participants also described ongoing efforts to renegotiate their identity over time.

##### Subtheme 3.1: Changing self-image

*Facilitator:* RA formed participant’s sense of self and how they were perceived by others. Many resisted being defined by their condition, taking pride in sustaining an identity as a person rather than a patient, particularly in work and social contexts. This promoted confidence and engagement with activities that might reduce SB.

*Barrier:* Tension existed between wanting to ‘fit in’ and the visibility of RA symptoms, i.e. hand deformities. Reduced confidence, stigma and societal perceptions of RA, such as it being viewed as an ‘old person’s disease’ discouraged participation in physical activities, indirectly increasing SB. Participants reconciled previous capabilities with current limitations, while lifestyle changes imposed by RA led to avoidance of activities participants once enjoyed and a sense of loss of identity.

##### Subtheme 3.2: Emotional consequences

*Facilitator:* SB carried complex emotional meaning that could be actively shaped. Rest was necessary for self-management, with self-compassion legitimizing SB, while fatigue-driven inactivity evoked guilt and loss of control. Some participants reframed sedentary periods as purposeful, by engaging in small activities, or redefining ‘achievement’, (e.g. stretching/walking), supporting wellbeing.

*Barrier:* Feelings of frustration, embarrassment, shame and guilt often intensified SB. Societal misunderstanding, and invisibility of symptoms amplified emotional strain when pain or limitations were minimized by others. Participants sometimes used SB strategically to conceal limitations, balancing physical comfort with social protection.

##### Subtheme 3.3: The social cost of RA

*Barrier:* RA shaped participants social engagement, leading to withdrawal, avoidance or isolation. Identity was affected by social consequences of being unable to participate in ways that aligned with how participants wished to be seen. Practical needs to sit, or manage fatigue, disrupted social interaction. For some, isolation stemmed from strained relationships, as friends grew impatient with the demands of RA. Judgement and social positioning reinforced sedentary patters.

## Discussion

This qualitative study investigated how SB is conceptualized by people with RA and identifies determinants of SB that go beyond well-recognized symptom-related barriers. Psychological and socio-cultural dimensions—including understanding, attitudes, beliefs, self-regulatory skills, social positioning and symptom-behaviour negotiations—offered great insight into the experiences of SB. This study addressed why people with RA engage in SB, how they felt about it and what internal and external determinants can facilitate or hinder SB. A major strength of this study was the involvement of PPI contributors in the development of the interview guide, incorporating perspectives that ensured questions were relevant, understandable and sensitive to the realities of people living with RA. These findings inform directions for developing SB interventions for people living with RA, with implications discussed below.

### Clarify SB vs PA in intervention messaging

The interpretation and understanding of SB varied in degrees of conceptual accuracy. Motivations were often expressed as wanting to ‘move more’, ‘be active’, or ‘exercise’, rather than specifically reducing SB. Findings from systematic reviews concur with our findings, identifying that older adults did not recognize the difference between SB and physical inactivity [27]. This conflation highlighted an important conceptual gap: while physical inactivity and SB are related, they are distinct behavioural constructs, which require different approaches to intervention. Future interventions should explicitly differentiate SB from PA through educational models and tailor behavioural strategies that target prolonged sitting and low-movement periods specifically.

### Ground interventions in patients’ motivations with meaningful activities

Active hobbies, (e.g. gardening, walking) promoted movement, but sedentary hobbies, (e.g. reading, bridge, social meals) were highly valued for enjoyment, identity and social connection. This challenges the view that SB is uniformly harmful [28]. Our findings concur with previous research demonstrating that sitting can have restorative value or enable social functions across populations [29] and hobbies benefit mental health management in chronic illness [30], socialization [31], self-identity and perceived physical health in people with arthritis [32]. Further, activity sustained valued identities. independence and quality of life, supported by previous literature [33].

Interventions should recognize the psychological and social value of sedentary hobbies, strategies could incorporate light movement (e.g. movement prompts between chapters of a book or standing while speaking on the phone), and distinguish between meaningful SB from symptom-driven inactivity.

Additionally, emphasizing quality of life and identity preservation may be stronger motivators for behaviour change, rather than abstract health benefits. Framing the reduction of SB as a practical tool for achieving personally meaningful outcomes may enhance engagement and adherence. Values-based and goal-directed approaches, promoting autonomy-supportive environments that are flexible in accommodating illness realities may also be useful. Given the alignment with Self-Determination Theory (SDT) [34, 35], which includes intrinsic motivation (enjoyment of hobbies), identified regulation (recognition of the health benefits) and integrated regulation (values such as independence and social connection), SDT appears a suitable behaviour change theory to address SB in this population.

### Design for symptom variability and the ‘flare cycle’

RA symptoms and flares were key determinants of SB across all themes, consistent with prior findings [20, 36, 37]. Symptom unpredictability influenced motivation and activity, with self-management in RA described as cyclical [38]. Participants used pacing—balancing activity with rest—highlighting the dynamic interplay between symptoms, functional capacity and behaviour.

Future interventional research may benefit from artificial intelligence, which presents opportunities for real-time, tailored support using symptom tracking, activity levels and geolocation data. Just-in-time Adaptive Interventions (JITAIs) have shown promise in PA [39] and reducing SB in obesity [40]. In RA, JITAIs could prompt movement during low-symptom and sedentary periods or support restorative rest and pacing guidance during flares. Such flexible approaches may better align with symptom variability than fixed activity goals, supporting adaptive, patient-centred SB management. Such interventional research must be co-designed with key stakeholders [41] and feasibility tested to ensure such approaches are acceptable and responsive to the needs of people with RA.

### Target environmental and contextual levels, not only individual behaviour

In addition to individual level factors, participants described structural and environmental factors that either hindered or enabled reduction in SB. Thus, interventions must also consider interpersonal and environmental level determinants, as per the socio-ecological model (SEM) [42]. For example, family and peers were described as both facilitators and barriers to activity, and social perceptions shaped confidence and willingness to engage in activity. Researchers should pay attention to these factors when developing interventions to reduce SB in RA, as multi-level interventions may be more effective than those targeting only individual level determinants of SB.

### Address identity change, emotional consequences and isolation

Identity disruption, functional loss, guilt, shame and stigma all shaped SB, suggesting interventions should include psychological support. Higher self-efficacy is associated with greater persistence and engagement in health behaviours [43] and greater goal achievement, acceptance of illness, problem-solving, physical activity participation, and improved physical and psychological health outcomes in chronic illness [44]. Self-efficacy can be strengthened through behaviour change techniques which deliver mastery experiences, vicarious learning, social persuasion and improved interpretation of physiological states in arthritis [45].

Self-efficacy can also be strengthened through resilience—i.e. adapting to stress, using individual and environmental resources [46]. In this study, resilience was reflected through (a) cognitive approaches (i.e. positive mindset/acceptance), (b) environmental modifications (i.e. assistive devices/home adaptations) and (c) proactive planning (i.e. meal preparation/accessibility checks). Given that psychosocial resilience factors are modifiable [41], resilience has an adaptive capacity that can be improved by future interventions.

### Limitations

This study has several limitations. First, participants were recruited through opportunistic and snowball sampling. The majority of interviews were also conducted online, which may not provide the same depth or richness of data compared with face-to-face interviews, due to less interpersonal dynamics that may be apparent in an online setting. Experiences of SB may differ according to RA characteristics (e.g. disease activity, physical function, duration since RA diagnosis). These characteristics were not collected in this study. However, our focus was on participants’ overall experiences of SB since diagnosis, for which point-in-time of disease status are less relevant given the fluctuating nature of RA; we did, however, capture how SB shifted in response to such changes. It is possible our results may not represent the broader population of people living with RA in terms of RA features and socio-demographics. Nonetheless, the qualitative data uncovered that our participants did represent a range of RA severity, disease activity, disease duration and sociodemographic variables (e.g. age, sex, employment status). Volunteer bias may also exist whereby, those who are more interested in SB and movement may have agreed to participate in this study, meaning perspectives of individuals who are less motivated to reduce SB—who may experience added barriers to movement, or do not perceive their SB as potentially problematic may be underrepresented. However, results offer important, in-depth insights into the complex nuances existing in regards to the lived experience of SB in RA, which are critical for the development of interventions that are effective and engaging.

## Conclusions

To conclude, interventions should clearly distinguish SB from PA, accommodate symptom variability and align recommended behavioural changes with personally meaningful goals. However, participant accounts did not always distinguish between influences on SB from those shaping PA, or movement more broadly, meaning that some of the identified determinants may reflect more general movement behaviour, rather than SB specifically. Approaches that support autonomy, recognize identity disruption and leverage existing adaptive strategies may be particularly effective. By framing reduction in sedentary time as a practical route to maintaining participation in valued life activities, future interventions may be better positioned to enhance engagement, relevance and sustainability.

## Supplementary Material

rkag137_Supplementary_Data

## Data Availability

The data underlying this article cannot be shared publicly due to the potentially identifiable and sensitive nature of the qualitative interview data, even following anonymization, and to protect the privacy and confidentiality of study participants. The data will be shared upon reasonable request to the corresponding author.

## Notes

*Disclosure statement*: The authors have declared no conflicts of interest.
