# Supplementary material for: Sedentary behaviour in rheumatoid arthritis—barriers and facilitators, a qualitative analysis
Source: Rheumatol Adv Pract. 2026 Oct 30;10(4):rkag137. doi: 10.1093/rap/rkag137 (PMC13742276; doi:10.1093/rap/rkag137)
Supplement: Supplementary file 1 [file rkag137_supplementary_data.zip › 26-143 Supplementary Data S2.docx]

**Supplementary Data S2. Interview guide**

| 1. **Knowledge of sedentary behaviour:** | |
| --- | --- |
| Can you tell me a little bit about what you understand about sedentary behaviour? | |
| ***IF THEY KNOW WHAT SB IS***  **Probes:**   - Where have you heard about sedentary behaviour (e.g. friends, family, news, doctor) - What is your understanding about sedentary behaviour in regard to your health/ in regards to your arthritis? - If believe good/bad then ask WHY/ what are the reasons you believe spending time sat down is good/bad for you? | ***IF THEY DON’T KNOW WHAT SB IS***  **Probes:**   - Sedentary behaviour can be explained as sitting behaviours, for example, watching TV, reading the news or driving. - Based on what I have just said, what is your understanding about these sitting behaviours in regard to your health/ in regards to your arthritis? |
| 2. **Lifestyle and interests:** | |
| Can you describe to me what a typical day looks like for you? From getting up in the morning, to going to bed at night. | |
| ***IF CLAIM TO BE SEDENTARY***  **Probes:**   - You mentioned you do X, what is the reason you do X?   - Do you do X out of necessity/ as a reward after completing a demanding activity/ for rest? - How do you feel when you do X? - When you have done X, what makes you “get up and go”? (Only relevant if SB is viewed as rest/necessity) | ***IF CLAIM TO BE NON-SEDENTARY***  **Probes:**   - What motivates you to be more on your feet and spend less time sitting down? - What type of activities do you do throughout the day that keep you on your feet? - Have you always been non-sedentary/How have things changed, if at all, since being diagnosed? - What type of physical exercise do you do? |
| - Does your daily routine differ according to how your RA feels e.g. if you are experiencing a flare? - Does your daily routine differ depending on your mood/e.g. if you are feeling low or anxious? | |
| 3. **Attitudes and beliefs about sedentary behaviour:** | |
| I would like to understand a bit more about your thoughts and feelings about sedentary behaviour. How has your upbringing or your culture, if at all, influenced your lifestyle and the way you view sedentary behaviour? | |
| **Probes:**   - How have your family values influenced your behaviours in regards to sedentariness or physical activity? - How has your culture influenced your behaviours in regards to sedentariness or physical activity? - What are your motivations to being less sedentary? - Have your feelings changed over the years towards sedentary behaviour? - Are you the same person you used to be since receiving your diagnosis? - Has your confidence in your abilities changed since receiving a diagnosis/ If yes: HOW does your confidence influence your day to day activities? - Does your confidence vary day by day – if so, what influences your confidence? | |
| 4. **Mental Health** | |
| Now, I would like to understand a bit more about your thoughts and feelings in general. Can you describe to me how your mental health is generally and how it may vary from day to day? | |
| **Probes:**   - Can you describe to me your level of independence on a typical day? How satisfied are you with this? - Can you describe to me how often, if at all, you feel anxious or gloomy? What would typically bring these feelings on? - Can you describe your relationships and how satisfied you are with the quality of these relationships? - What are your coping mechanisms for your RA/ How do you cope with flare ups or low days? - Do you think or worry at all about your physical health? If YES: What sorts of thoughts do you have? | |
| 5. **Barriers to reducing sedentary behaviour** | |
| Is there anything that makes it harder for you to reduce your sedentary behaviour? | |
| **Probes:**   - If something is identified, WHY do you think this makes it harder/ HOW does it hinder/stop you from reducing your sedentary behaviour? - Are there barriers within you living circumstances that make it harder? - Are there barriers within your work environment that make it harder? - Are there any social influences that make it harder for you to engage in less sedentary activities?   *If earlier mention of high disease activity (flare):* How does a flare influence the amount of time you spend sedentary compared to a more normal day?  *If earlier mention of mental health influences:* How does your mood affect the amount of time you spend sedentary/ to what extent can you attribute sitting time to your mental health? | |
| 6. **Facilitators to reducing sedentary behaviour:** | |
| Is there anything that makes it easier for you to reduce your sedentary behaviour? | |
| **Probes:**   - If something is identified, WHY do you think this makes it easier/ HOW does it help you to reduce your sedentary behaviour? - Are there things within your living circumstances that make is easier? - Are there things within your work environment that make it easier? - Are there any social influences that make it easier? - E.g. Do you have any social influences to support or remind you to engage in non-sedentary behaviours? - Is there anything that encourages you to engage in a less sedentary lifestyle?   *If earlier mention of low confidence earlier:* What do you think would be useful to build your confidence?  *If earlier mention of mental health influences:* What things could help your mental health on days where you feel low?   - Is there anything you can think of that you don’t currently have, that would make it easier for you to reduce your sedentary behaviour? | |
